# The Effect of Childhood Adversities and Protective Factors on the Development of Child-Psychiatric Disorders and Their Treatment

**DOI:** 10.3389/fpsyg.2018.02226

**Published:** 2018-11-15

**Authors:** Egon Bachler, Alexander Frühmann, Herbert Bachler, Benjamin Aas, Marius Nickel, Guenter Karl Schiepek

**Affiliations:** ^1^Institute for Synergetics and Psychotherapy Research, Paracelsus Medizinische Privatuniversität, Salzburg, Austria; ^2^Institut für Psychoanalyse und Familientherapie, Salzburg, Austria; ^3^General Medicine, Innsbruck Medical University, Innsbruck, Austria; ^4^Institute for Psychology, Ludwig-Maximilians-Universität München, Munich, Germany; ^5^Department of Psychiatry, Medical University of Graz, Graz, Austria

**Keywords:** protective factors, multi problem family, childhood adversities, outcome research evaluation, family therapies

## Abstract

**Context:** Families with high rates of childhood adversities (CAs) (multi problem families, MPF) have an increasing importance in public health-policy.

**Objective:** The present study addresses the relationship between risk- and protective factors and the severity and treatment-outcome of mental disorders.

**Setting:** Family-therapeutic home-based treatment for MPF. We examined a clinical sample (*N* = 1031) of children between the age of 4 to 17, and a non-clinical sample of 148 children. We hypothesized that of all children of the clinical group have a predominance of risk factors and a higher number of psychopathological symptoms. Furthermore, we hypothesized that children with a predominance of protective factors benefit stronger from psychotherapy.

**Main Results:** In the clinical sample, most children met the criteria of a psychopathological diagnosis (95.7%, as compared to 21.6% in the non-clinical sample) and showed significant higher rates of CAs and significant less protective factors as compared to the non-clinical sample. The clinical group showed a significant reduction of psychopathological symptoms and benefited equally well from treatment. The number of risk factors was a significant predictor for a child from the non-clinical sample to meet the criteria of a psychopathological diagnosis, while the number of protective factors significantly predicted the absence thereof.

**Conclusion:** Children and adolescents with high scores of CAs show significant associations with child psychiatric symptoms (*d* = 0.35; including all ICD-diagnosis such as, e.g., Asperger Syndrome, ADHD etc. with a higher rate of genetic etiology). Early life stressors, however, do not trigger an irreversible fate, as psychotherapy with young people with high numbers of risk factors does help to reduce psychopathological symptoms significantly (range of five outcome parameters: *d* = 0.31–0.72).

## Introduction

Epidemiological data show that up to 20% of children and adolescents show mental disorders (Belfer, 2008). The prevalence of neglect, maltreatment, deprivation in childhood in Germany is estimated with about 10–15% (Reichl et al., 2014), higher rates were found in multi problem families (MPF; Belfer, 2008), which leads to an increased risk of vulnerability for children (Egle et al., 2004; Bachler et al., 2014). High-risk families not only meet many criteria of family adversity indices, but also show low treatment compliance and low relational functioning of the families (Bachler et al., 2017). MPF exhibit structural, dynamic, and social characteristics that could lead to an increased risk of vulnerability and implement and test treatment procedures that help adults and children of such multi-problem or high-risk families. Typically, families in such problematic situations exhibit disorders in parental relationship behavior, have a lower socioeconomic status (SES), and are associated with conditions of deprivation (Woolfenden et al., 2001; Witkiewitz et al., 2013). Low SES has been shown to be linked to parental behavior as a moderating and mediating terms of being less engaged, less parental monitoring and a less enriched environment, thereby exerting negative attachment-related effects to child development (Bornstein and Bradley, 2003). There is an extensive research about mediating and moderating factors in the development of psychopathological symptoms (Luo and Waite, 2005; Lund et al., 2011; Cabaj et al., 2014; Rasmussen et al., 2014).

These problems sometimes run across many generations in the families, which eventually creates a lack of trust into governmental and municipalities’ services and a belief that the life’s problems are an inevitable fate. Therefore, MPF form a clientele that is sometimes referred to as “hard to reach,” even though it has been shown that psychotherapy is effectively possible (Curtis et al., 2004). Untreated, multi-generational psycho-social problems have an impact on and interaction with education, SES, general health, quality of life, number of children and more, and therefore have been shown to form a high economic burden to societies (Wittchen and Jacobi, 2005). Detection and treatment of MPF is thus ethically and financially of high importance to social policy and the general public, in order to not allow these networks of childhood adversities (CAs) forming it to an irreversible damage as measured by psychiatric diagnoses/symptoms.

Kessler et al. (2010) undertook the approach of relating 12 CAs – grouped into clusters of interpersonal loss, parental maladjustment, maltreatment and others – with the onset of 20 DSM IV disorders in a cross-national study. They found that in high-income as well as in low-income countries, there was an almost equal chance of 38.4% (and 39.1%) to report one or more of the CAs. The presence of one or more CAs accounted for 28.2% of all DSM-IV diagnoses over all countries, and were significantly correlated with an increased risk for DSM-IV disorder (Klasen et al., 2015). Slopen et al. (2014) examined the influence of high and chronic exposure to CAs at different developmental periods in childhood on internalizing and externalizing symptoms, as well as other health related parameters (weight, blood pressure) of adolescents or adults (Edwards and Hans, 2015). They showed that onset and continuance of cumulative childhood adversity are influencing mental and physical health risks in general. Similarly, it has been shown that children or adolescents with 4 or more CAs were 7.3 times more likely to display depression, anxiety, affect regulation problems, or substance abuse (Putnam et al., 2013). Besides forming general risk factors, CAs might also play a direct role in onset of specific disorders and specific symptoms (Bentall et al., 2014; Young and Widom, 2014), while it has also been found that severity of CAs plays a bigger role than their mere number (Schilling et al., 2008).

Research into the underlying mechanisms of cross-generational impact of CAs sketch a picture of interactions between nature and nurture. Cicchetti and Rogosch (2012) reported a different genotype between maltreated and non-maltreated children, which was associated to their individual level of resilience. The influence of bonding patterns has been demonstrated in several studies (Haushofer and Fehr, 2014; Reichl et al., 2014). The neuropathophysiology of psychosocial stress and stress responsivity has, e.g., been linked to the HPA axis, as well as neurogenesis and the development of functionality and morphology of the brain (Bick et al., 2012). Kim et al. (2013) revealed the influence of childhood poverty in low SES families and CAs on amygdala and prefrontal cortex dysregulation. This proven shift in the subcortical mode is a result of individually different susceptibility to qualities of environments through genetic factors as Bakermans-Kranenburg and van Ijzendoorn (2006) showed for the DRD4 dopaminergic system, behavior problems and parenting. Environments (e.g., parenting) are steering epigenetic processes and children’s behavior is forming parenting (Hartman and Belsky, 2016). Taken together, there is empirical evidence that CAs and also protective factors are integrated into the interacting levels of behavior, environment, and gene-expression, as it is conceptualized in the model of genetic differential sensitivity to social environment (GDSE, Hengartner et al., 2013; Mitchell et al., 2013; McDonald et al., 2016; Moore and Depue, 2016).

The existing literature and research is, however, thin on the interaction between protective factors and risk-factors and especially their influence on psychotherapy in form of increased number of symptoms and effectiveness of treatment (Andershed and Andershed, 2015). The present research therefore aimed at replicating earlier results, that children and adolescents from a non-clinical sample have lower risk factors and more protective factors as compared to a clinical sample for a German-Austrian setting. Furthermore, we tested the hypothesis that the number of risk- and protective factors (and their combination) predicts the presence and absence of psychopathological diagnosis in a non-clinical sample. In addition, we addressed the respective relationship between CAs and protective factors and symptomatic burden.

## Materials and Methods

### Participants

TAF-treatment in the clinical sample is initiated through the proposal of the regional youth services (Salzburg, Upper Austria and South East of Bavaria-Germany). The indication is done after a first diagnostic and selective-indicative clearing. All 1031 families for whom the treatment began in 2008 or later but only if the index child was older than 4 years were included in the study (consecutive sample). The data on the children and adolescents in the comparative sample (*n* = 148) were collected at a grammar school in southern Germany. The drop out quote of the clinical group was 17% (failed compliance). The treatment length 21.6 months (mean).

The clinical sample of the present study consisted of all patients/families, entering TAF in the years between 2008 and 2017. Diagnostic clearing cases cover patients that were placed in new families, entered outpatient psychotherapy or suffered of severe psychopathology.

An overview of socio-demographic data, ICD-10 diagnoses and symptom-score (pre-post) can be seen in Table 1.

**Table 1:** Demographics, diagnostics, and pre-/post-symptom scores.

	Clinical group					Non-clinical	
	Pre		Post		ES^∗^		
	*N*	*M* (*SD*) or %	*N*	*M* (*SD*) or %		*N*	*M* (*SD*) or %
Age	1028	15.7 (4.9)				148	11.8 (3.2)
Sex	1028	46.0%♀				148	52% **♀**
Treatment length (month)	1031	21.6 (15.2)					
SES	1030	3.21 (1.24)	1030	2.94 (1.2)			
Drug addiction parent(s)	902	26.9%					
Uneducated (FAI1)	1004	31.3%					
Disharmony/Single parents	1009	76.9%					
ICD-diagnosis-total	913	95.7%	897	60.2%		148	21.6%
Emotional disorder	*n* = 455	49.8%	*n* = 281	31.3%		148	13.5%
Dysregulation disorder	*n* = 136	14.9%	*n* = 55	6.1%		148	1.4%
ADHD	*n* = 207	22.7%	*n* = 130	14.5%		148	6.8%
Special diagnoses	*n* = 76	8.3%	*n* = 74	8.2%		148	0%
Severity of problems (MPI)	1009	12.5 (9.35)	1002	7.73 (7.69)	0.61	148	4.1 (3.9)
Family adversity index	1031	2.33 (1.11)	1031	2.05 (1.12)	0.34		
Caregiver (GAF)	1031	61.2 (15.6)	1031	66.3 (16.3)	0.44		
Child (CGAF)	754	57.2 (15.5)	754	67.0 (16.4)	0.72		
Relation (GARF)	805	58.2 (18.0)	805	68.2 (18.9)	0.54		

*^∗^ES, effect size Cohen’s d, N = valid cases. TAF, Therapeutic Ambulant Family Care; SES, socioeconomic status; FAI, family adversity index; GAF, Global Assessment of Psychosocial Functioning; CGAF, childrens’ GAF; MPI, Mannheim Parental Interview; GARF, Global Assessment of Relational Functioning.*

### The Treatment Method TAF

TAF (therapeutic, outreach intervention) is a disorder-oriented, therapeutic, intervention for MPF. It constitutes an integrative form of family therapy (FT) for MPFs and integrates family-therapeutic interventions and elements of structural psychotherapy. TAF incorporates the various common principles for treatment of structural psychotherapy (for the improvement of ego-structural competencies) that were identified by the task force of the APA Division 12: strong working alliance, therapist’s ability to repair alliance-ruptures, collaboration on goals, and a high level of therapist activity (Critchfield and Benjamin, 2006).

### Instruments of Measurement

All instruments of measurement used in this study are part of a standardized in-house manual (TAF-DOK). Some of the instruments of TAF-DOK are only applicable to children under the age of 4 years. A total of 118 cases were therefore not considered in the clinical group because we did not have access to a kindergarten.

#### Mannheim Parental Interview (MPI)

The MPI (Esser et al., 1989) is a structural, standardized, clinical interview for therapists to assess the severity of the mental disorder of children, adolescents, and primary attachment figure. The interview is divided into three parts: the parents’ and child’s demographic and social statistics, child and adolescent psychiatric symptoms and socio-familial conditions/and important life events. The 37 questions regarding child and adolescent psychiatric symptoms are leading through clearly stipulated criteria and scores of severity to a cumulative child-psychiatric symptom-score and different ICD-10 diagnoses (non ICD-10 diagnose; ICD-10 F92-98 mood, anxiety, and attachment disorders; ICD-10 F 90 ADHD; ICD-10 60 personality disorders, ICD-10 F 91 externalizing symptoms and maladaptive behavior; and F84-89 special diagnoses ICD-10) and comorbidities. The interrater reliability is 0.71–1.0, i.e., the kappa coefficient of concurrence of the diagnoses was determined as *r* = 0.71 (percentage of concurrence 79% between the clinical, professional opinions).

#### Family Adversity Index (FAI)

The FAI was developed by Rutter and Quinton (1977). It measures five family-related CAs of chronic disharmony in the family, a low SES, small living quarters, parental criminality, and mental disorder of the mother. Reliability is given as 0.65, and validity in the range of 0.66 to 0.70 (Rutter and Quinton, 1977).

#### Adverse and Protective Life Events

Egle et al. (2004) presented a meta-analytic overview of empirically found protective and adverse life events. The results of this meta-analysis were used to create a screening questionnaire, which includes 22 adverse live events. CAs were assessed dichotomously.

We generated five Clusters of CAs: *Cluster 1a (SES-Resources):* low SES, poor schooling of parents, unemployment, large family and very narrow living room, single parent, contacts with institutions of social control; *Cluster 2a (violence):* crime or antisocial behavior of parents, chronic disharmony in the family, authoritarian paternal behavior; *Cluster 3a (attachment related CAs):* maternal occupational activity in the first year of life, insecure attachment behavior after 12/18 months of age, loss of parent, divorce, separation of parents, frequently changing early reference person, improper contacts with peers, age distance to the nearest siblings younger than 18 months, prolonged separation from the parents during the first 7 years of life, male sex; *Cluster 4a (physical or mental illness):* mental disorder of the father or mother, severe physical illness of the father or the mother, chronically ill sibling; *Cluster 5a (single or cumulative trauma):* sexual abuse and physical maltreatment and emotional neglect. Similarly we recorded eight protective life events: *Cluster 1p* (social resources): good relationship with at least one primary caregiver, secure attachment behavior, extended family, compensatory parents relationship, good replacement milieu after an early mother loss, social promotion (e.g., school, church, reliable support reference person); *Cluster 2p* (individual resources): above average intelligence, robust active temperament, self-efficacy.

#### Childhood SES (SSE-TAF)

*SSE-TAF* (social self-sufficiency) is a validated (interrater reliability 0.73) part of the TAF documentation (TAF-DOK) and tries to document the social self-preservation ability of the family with one item (Bachler et al., 2014). The SSE TAF measures the ability to work and is a is based on a 5-tiered Likert scale.

#### Global Assessment of Functioning Scale for Adults and Children (GAF, CGAS)

Global Assessment of Psychosocial Functioning is used as measurement for scaling the individual competence-related and psychosocial functional level. It includes interpersonal and employment abilities and represents a one-dimensional depiction of a patient’s psychosocial functioning level (Soderberg et al., 2005). The rating ranges between 0 and 100. The interrater reliability has been reported with 0.74. A score of ≤50 indicates severe limitations.

#### Global Assessment of Relational Functioning (GARF Scale)

The GARF scale was used to rate the psychosocial functioning level of the families (Stasch and Cierpka, 2006). The GARF detects three dimensions: (a) problem solving; (b) organization; and (c) emotional climate. The scaling occurs between 0 and 100. The interrater reliability is reported with 0.72, the Cronbach alpha with 0.91, and the generalizability coefficient (GC) with 0.93.

### Procedure

The data of the clinical sample was collected by the respective therapist of a family during the first 3 months of treatment. The non-clinical sample was drawn from two schools in Bavaria (Elementary School and High School), using the same methods as in the clinical sample. The non-clinical sample was matched (age and gender) according to the characteristics of the clinical group. For the statistical analyses (calculating mean, standard deviations, independent samples *t*-test, logistic binary regression, *Pearson’s correlation coefficient*, and linear regression analysis) IBM^®^ SPSS^®^ Statistics Premium GradPack 23 for Windows has been used.

There is no potential conflict of interest. This study was carried out in accordance with the recommendations of WMA Declaration of Helsinki – Ethical Principles for Medical Research Involving Human Subjects. All subjects gave written informed consent in accordance with the Declaration of Helsinki. The investigation has been approved by the Ethics Committee of our Institutions (Institute for Psychoanalysis and Family Therapy Salzburg Austria).

## Results

### Prevalence Rate of Psychopathological Diagnosis

In the clinical sample 95.7% of the cases met the ICD-10 criteria for at least one psychopathological diagnosis, compared to 21.6% in the non-clinical sample.

### Risk- and Protective Factors

The mean number of CAs was 7.81 (*SD =* 3.18) for the clinical sample and 1.83 (*SD =* 2.08) for the non-clinical sample. Therefore, the clinical sample met on average 35.49% (*SD =* 14.48%) of 22 CAs, while the non-clinical sample did so for 8.35% (*SD =* 9.47%). Tested with an independent samples *t*-test, this constitutes a significant difference [*t*(1155) *=* 22.12, *p* < 0.001, *d =* 2.27], meaning that kids in the clinical sample had significant more CAs.

**FIGURE 1:**
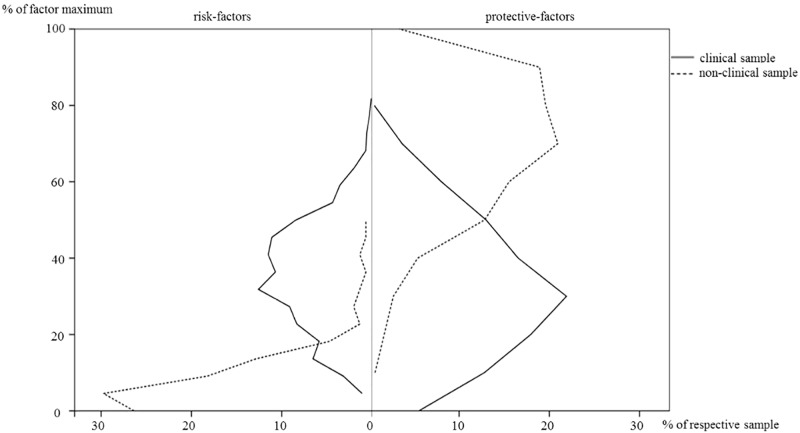
Percentages of corrected risk- and protective-factors in the clinical and non-clinical sample.

As can be seen on the left side of Figure 1, children in the clinical sample (solid line) were exposed to more risk factors as compared to the non-clinical sample (dashed line). In addition – as scan be seen on the right side of Figure 1 – persons in the clinical sample grew up with significantly less protective factors [clinical: *M* = 32.41%, *SD =* 18.1%; non-clinical: *M* = 69.53%, *SD =* 17.63%; independent samples *t*-test: *t*(1155) = -23.37, *p* < 0.001, *d* = -2.08].

The three most prevalent CAs in the non-clinical sample were divorce or separation of parents (14.2%), employment of mother during first year after childbirth (21.2%) and a low SES (10.9%).

Cluster 5a (single or cumulative trauma): sexual abuse, physical maltreatment, and emotional neglect.

### Relationship Between Psychopathology and Risk-/Protective Factors in a Non-clinical Sample

To test the relationship between the risk-/protective factors with the presence of a psychopathological diagnosis, we conducted a logistic binary regression. Both, CAs (*B* = 5.06, *exp b* = 158.01, *p* = 0.017) and protective factors (*B* = -0.34, *exp b* = 0.03, *p* = 0.006), turn out to be significant predictors for the existence/absence of a psychopathological diagnosis in the non-clinical sample [*X*^*2*^(1) = 18.0, *p* < 0.001; *R*^*2*^ *=* 0.177 (Nagelkerke, 1991), 0.112 (Cox and Snell, 1989)]. These results suggest that in a normal population of schoolchildren, protective factors make it significantly less likely to have a psychopathological diagnosis, while risk factors increase that chance significantly. In our non-clinical sample, children with a negative ratio of protective to risk factors had a 9.4% chance of meeting criteria for an ICD-10 diagnosis, while none (0%) of the kids with a positive ratio (more protective factors) did.

### Treatment Effect in the Clinical Sample

**FIGURE 2:**
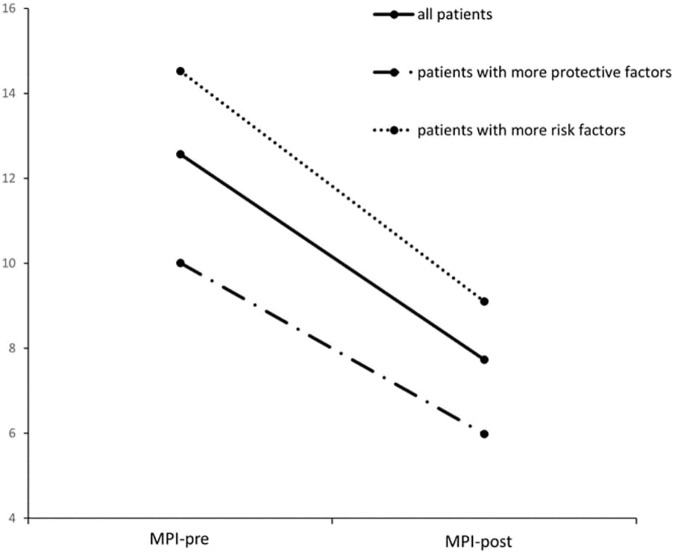
Risk and protective factors and their relationship to child psychiatric symptoms.

For the clinical sample, all psycho-social post treatment scores were significantly lower as compared to before treatment. The psychopathological symptoms as measured by Mannheim Parental Interview dropped from 12.57 (*SD* = 9.35) to 7.73 (*SD* = 7.69) after treatment, as can be seen in Figure 2. Tested with a paired samples *t*-test, that constitutes a significant reduction in psychopathologic symptoms [*t*(1000) = 18.94, *p* < 0.001; *d* = 0.61], even though the sample includes high rates of diagnoses like adolescent personality disorders and Asperger-syndrome with lower therapeutic variability. The family adversity index (FAI) significantly dropped from 2.33 (*SD* = 1.11) to 2.05 [*SD* = 1.12; *t*(1030) = 11.19, *p* < 0.001, *d* = 0.35]. The ability for social self-preservation (SSE-TAF) improved from 3.21 (*SD* = 1.24) to 2.94 [*SD* = 1.20; *t*(1029) = 10.05, *p* < 0.001, *d* = 0.31]. The three assessments of global functioning in terms of caregiver (GAF) [*M*pre = 6.12, *SD* = 1.56, *M*post = 6.63, *SD* = 1.63; *t*(1030) = -14.52, *p* < 0.001, *d* = -0.45], index child caregiver (CGAF) [*M*pre = 5.72, *SD* = 1.55, *M*post = 6.70, *SD* = 1.63; *t*(753) = -19.83, *p* < 0.001, *d* = -0.72] and relational functioning caregiver (GARF) [*M*pre = 5.82, *SD* = 1.80, *M*post = 6.82, *SD* = 1.89; *t*(804) = -16.01, *p* < 0.001, *d* = -0.54] all also improved significantly.

### Risk-Factors and Their Relationship to Psychopathological Symptoms

The number of risk factors was significantly correlated to all measures related to psychological problems in the clinical sample (MPI: *Pearson’s r* = 0.17, *p* < 0.001; FAI: *Pearson’s r* = 0.46, *p* < 0.001; GAF: *Pearson’s r* = -0.3, *p* < 0.001; CGAF: *Pearson’s r* = -0.21, *p* < 0.001; GARF: *Pearson’s r* = -0.25, *p* < 0.001) and also in the non-clinical sample (MPI: *Pearson’s r* = 0.32, *p* < 0.001). The hypothesis that the higher the number of CAs – and therefore risk-factors – coincides with a higher number of psychopathological symptoms, was confirmed by a linear regression analysis [*F*(1,1007) = 30.29, *p* < 0.001]. The parameter of the risk-score significantly predicted the number of psychopathological symptoms [*t*(1007) = 11.24, *p* < 0.001]. With a *R*^*2*^ = 0.03 and the value *b* = 11.02, these results can be interpreted in a way, that for each two additional risk-factors, we can predict an increase of one point on the psychopathological symptom-score at the beginning of treatment, as measured by the MPI.

We did not find evidence for the hypothesis that higher risk-scores have a predictive value for the treatment effect [*F*(1,999) = 2.04, *p* = 0.154], suggesting that treatment is as effective for patients with high risk as it is for patients with only a few or no risk factors.

### Protective-Factors and Their Relationship to Psychopathological Symptoms

The protective-factors were significantly correlated to the number/severity of psychological problems, in the opposing direction of the risk-factors for the clinical sample (MPI: *Pearson’s r* = -0.26, *p* < 0.001; FAI: *Pearson’s r* = -0.18, *p* < 0.001; GAF: *Pearson’s r* = 0.23, *p* < 0.001; CGAF: *Pearson’s r* = 0.32, *p* < 0.001; GARF: *Pearson’s r* = 0.23, *p* < 0.001) and also in the non-clinical sample (MPI: *Pearson’s r* = -0.26, *p* < 0.001), constituting the exact complementary result of the risk factors. We tested the hypothesis that a higher number of protective factors predicts lower number of psychopathological symptoms. A linear regression analysis revealed that patients’ number of protective factors significantly predicted the pre-treatment scores of MPI [*F*(1,1007) = 73.31, *p* < 0.001]. The parameter of protective-factors with an *R*^*2*^ = 0.07 and *b* = -13.43 significantly predicted [*t*(1007) = -8.56, *p* < 0.001] that for each protective factor the psychopathological symptom-score can be expected to be 1.3 lower on average, constituting a buffering effect of protective-factors.

The hypothesis that the number of protective factors also predicts the number of symptoms after treatment was met in the opposite direction as previously hypothesized [*F*(1,999) = 10.94, *p* < 0.001]. With an *R*^*2*^ = 0.01 and a *b* = 4.63, the linear regression analysis significantly predicted [*t*(999) = 3.31, *p* = 0.001] that for each additional protective factor, the treatment will reduce 0.46 less symptoms. This might be interpreted in the light of the results that patients with more protective factors had less symptoms to start with at the beginning of treatment and therefore less chance to reduce these, as can be seen in Figure 2 and is reported in the next paragraph.

### Effect of the Net-Score of Risk- vs. Protective-Factors

By subtracting the corrected risk-factors from the corrected protective factors, we separated the clinical sample into two groups of patients with predominance of risk-factors (risk-group, *n* = 556) and predominance of protective-factors (protective-group, *n* = 445), respectively.

As can be seen in Figure 2, the mean psychopathological symptoms of these two groups at beginning of treatment (risk-group: *M* = 14.58, *SD* = 9.53; protective-group: *M* = 10.07, *SD =* 8.48) differed significantly [*t*(1007) = 7.72, *p* < 0.001].

Even though both groups significantly reduce their psychopathology scores during treatment [risk-group: *M* = 9.16, *SD* = 8.23, *t*(555) = 14.53, *p* < 0.001; protective-group: *M* = 5.96, *SD* = 6.54, *t*(444) = 12.33, *p* < 0.001], there remains a significant difference between the two groups [*t*(999) = 6.69, *p* < 0.001] after treatment. The risk group – dotted line in Figure 2 – did show a trend for having a bigger total reduction of their symptoms [*F*(1,999) = 6.53, *p* = 0.011]. Taken together, patients with a higher number of CAs than protective factors showed more psychopathological symptoms before and after treatment, but had a tendency to reduce more of their symptoms, as compared to children that had more protective factors.

### Correlation of Clusters of CAs and PFs With SES, Symptom-Score at Begin and Reduction of Symptoms

As can be seen in Table 2, the different clusters of protective- and risk factors correlated differently with psychopathological symptoms at beginning of the treatment. The cluster of attachment related risk-factors show the strongest relation to the symptoms (*Pearson’s r* = 0.24, *p* < 0.001), with the other clusters of violence (*Pearson’s r* = 0.12, *p* < 0.001) and trauma (*Pearson’s r* = 0.13, *p* < 0.001) to be also significantly correlated.

**Table 2:** Bivariate correlation matrix of current stressors (socio-economic status and psychopathology), childhood adversities (risk-clusters), and protective clusters.

Pre-treatment measures	1	2	3	4	5	6	7	8	9	10
*Current stressors:*										
(1) SES-TAF		0.012	0.037	0.658*	0.195*	0.148*	0.165*	0.09*	-0.194*	-0.154*
(2) Symptomscore_pre (MPI)			-0.619*	-0.002	0.120*	0.235*	0.021	0.125*	-0.247*	-0.157*
(3) Symptomscore-reduction (MPI)				0.026	-0.009	-0.103*	0.016	-0.049	0.079‘	0.092‘
*Risk clusters:*										
(4) SES-resources					0.228*	0.253*	0.180*	0.130*	-0.172*	-0.099*
(5) Violence						0.169*	0.199*	0.298*	-0.219*	0.000
(6) Attachment							0.136*	0.150*	-0.253*	-0.147*
(7) Physical or mental illness								0.101*	-0.079‘	-0.054
(8) Trauma									-0.142*	-0.015
*Protective clusters:*										
(9) Social resources										0.281
(10) Individual resources										

*^∗^Significant at >0.01; ‘significant at 0.05; *N* = 1009 (symptomscore reduction *N* = 1001)*.

Both clusters of the protective factors are significantly negative correlated with the symptom-score at beginning of treatment (social resources: *Pearson’s r* = -0.25, *p* < 0.001; individual resources: *Pearson’s r* = -0.16, *p* < 0.001).

## Discussion

Multi-problem families are often faced with a cross-generational, self-sustaining network of problems, where the social, educational, occupational, physical, and psychological maladaptation of one generation increase the risk of the family’s next generation. In our study, a representative sample of 148 children and adolescents from two schools were faced with an average of 1.84 (8.35% of 22) childhood-adversities, while a clinical sample of 1009 kids were exposed to a significant higher number of CAs (7.81; 35.49%). These children not only grow up under circumstances of heightened risk, they also have a significant lower number of positive, protective factors at their disposal (clinical sample: 32.41%; non-clinical: 69.53%). Children that meet the criteria of a psychopathological diagnosis will have encountered a smaller SES, more violence in the family, attachment-difficulties, more psychological and physical illness of their family members and are more likely to have gone through a traumatic experience, while they have less individual and social resources to cope with these adversities. In such an environment, psychopathology possibly has better chances to evolve and persist.

In addition, not only the presence of a diagnosis is more likely, but also the number of symptoms is significantly positive correlated with different clusters of CAs (and negatively correlated with the two protective clusters) in the clinical sample as well as in the non-clinical sample. For each two more CAs encountered in early life, the symptomatic score increases on average by one point when at the beginning of treatment. Each protective factor reduces that score by 1.3 points.

These results clearly support the notion that growing up in a family with multiple problems forms a thread to future psychological health of a child, but that fate is not irreversible. When undergoing psychotherapeutic treatment, as in the present clinical sample, the average patients – independently of their adverse or beneficial circumstances – benefit significantly from therapy. The treatment effect did not depend on the severity of disorders. To get a deeper understanding of the role, CAs and protective factors play in therapeutic processes, we divided the group of all patients into two groups that either had a predominance of adversities or protective factors. The group with more risk factors displayed a higher symptom-score before and after treatment. However, this group presented itself with an equally strong reduction of symptoms after therapy. In fact, the symptomatic drop was almost stronger for the risk-group, which, however, has to be interpreted in the light of having started out with more symptoms.

## Conclusion

Combined, these results of therapeutic effectiveness shed a positive light on the question, whether the cross generational network of problems can be dismantled. Knowing that therapy even in severe circumstances is possible, not only demands public investment into therapy for the young person at hand, but is publicly even more beneficial, since it is likely to cut the cross-generational inheritance of adverse living conditions. That argument is supported by the comparison of a clinical sample with a non-clinical sample, where it could be shown that the chances for developing a psychopathology decreases with the decrease of CAs and increase of protective factors in early life.

The understanding of the etiology of psychological problems needs to take into account the framework of CAs and their interaction with protective factors. The individual risk of developing mental health problems is possibly caused by individual vulnerability, personal and family-related resources (PFs), as well as by the age, frequency, the duration and the accumulation of various risk factors. However, even when a kid grows up in bad circumstances and does develop psychopathological symptoms, therapy is possible and with high outcome values. Adaptive treatments can prevent disorder-related long-term effects and can decrease family related indices of CAs (FAI, *d* = 0.35). Our study showed that therapeutic home-based treatment with high structural and process quality can successfully treat groups with severe child-psychiatric disorders. But we need further research about treatment aptitude and “what works for whom” (Beutler et al., 2016).

Our data allow us to point out the importance of CAs × PFs interactions as a framework to explain individual differential susceptibility for environmental influences in G × E interaction. This will have relevance for preventive and curative interventions in child-protection, and health policy pay off, because the direct and indirect costs of CAs over the course of lifetime are very high (Masten, 2011). The costs of the developmental effects of serious child maltreatment exceeds 210.000$ per victim (Klika and Herrenkohl, 2013). Empirical research about these issues are important because the long-term effects of CAs are harmful and costly. We can summarize that the development of child psychiatric symptoms is determined by the complexity of a number of genetic, epigenetic, and different environmental influences (socio-familial, socioeconomic factors, and by CAs × PFs interactions) with non-additive influence at different stages of age.

Three important tasks can be derived from the presented results: First, families, neighbors, schools, social services and governments have to take action to prevent, screen and if necessary, act quickly on harsh conditions in and for families, for not allowing to have violence and rates of early life stressors dominate childrens’ life’s (Flouri et al., 2015; Bethell et al., 2016). Second, more empirical research has to be conducted, to better understand dysfunction specific resilience mechanisms and underlying gene–environment interactions. The regulating function of the HPA axis and in consequence long-term functionality of the brain is, e.g., just one mechanism, how early uninterrupted life-stressors might have a lasting influence (Bick et al., 2012). Third, generalizing research on groups has to be expanded by designs that allow a better understanding of which treatment might help whom and when the best. Therefore our empirical study is a contribution to treatment aptitude research (Beutler et al., 2016).

### Limitations

Even though we had three sources of data with high reliability to the assessment of the CAs (primary care giver, adolescents, and therapists), retrospective assessment has possible biases in terms of under-reporting (memory) or over-reporting (trying to please the interviewer). Furthermore, the study has a naturalistic design, the treatments were conducted as usual, with adherence check for the interventions. Therefore, causal interpretations, are, in a strict sense, not possible.

## Author Contributions

All authors designed the study and proofread the literature. BA was responsible for statistics. EB and AF collected the data. EB, HB, MN, and GS wrote the paper.

## Conflict of Interest Statement

The authors declare that the research was conducted in the absence of any commercial or financial relationships that could be construed as a potential conflict of interest.
